# The Influence of Femoral Proximal Medullary Morphology on Subtrochanteric Osteotomy in Total Hip Arthroplasty for Unilateral High Dislocated Hips

**DOI:** 10.1111/os.13039

**Published:** 2021-08-05

**Authors:** Yin‐qiao Du, Ling‐fei Guo, Jing‐yang Sun, Jun‐min Shen, Bo‐han Zhang, Zhi‐gang Jin, Yong‐gang Zhou

**Affiliations:** ^1^ Department of Orthopaedics Chinese People's Liberation Army General Hospital Beijing China; ^2^ Department of Orthopedics Northeast International Hospital Shenyang China

**Keywords:** Developmental dysplasia of the hip, Femoral medullary morphology, Subtrochanteric osteotomy, Total hip arthroplasty

## Abstract

**Objective:**

To evaluate the predictive values of femoral proximal medullary morphology for the use of subtrochanteric osteotomy (STO) in unilateral Crowe IV developmental dysplasia of the hip (DDH).

**Methods:**

Ninety four patients with unilateral Crowe type IV DDH (59 hips in STO group and 35 hips in the non‐STO group) between April 2008 and June 2019 were enrolled. All patients underwent THA using the Pinnacle acetabular shell, ceramic liner and femoral head, the S‐ROM stem with proximal sleeve. Three parameters on the standard anteroposterior hip radiographs were measured: the widths of medullary canals at 20 mm above the center of lesser trochanter (CLT)，20 mm below the CLT and the isthmus. Canal flare index (CFI), metaphyseal canal flare index (MCFI), diaphyseal canal flare index (DCFI) were calculated. A S‐ROM femoral stem was used in all patients during total hip arthroplasty (THA).

**Results:**

The CFI and DCFI in the STO group were lower than those in the non‐STO group. However, there was no statistical difference in MCFI between the two groups. The receiver operating characteristic (ROC) curves shown that DCFI had the highest area under the curve (AUC), at 0.885. This was followed by the CFI, which had an AUC of 0.847. The AUC of MCFI was 0.579. The optimal threshold for DCFI was 1.44, which lead to a sensitivity, specificity, positive predictive value (PPV), and negative predictive value (NPV) of 0.771, 0.898, 0.869, and 0.818, respectively. For CFI, the optimal threshold was 3.28, resulting in a sensitivity, specificity, PPV, and NPV of 0.829, 0.729, 0.878, and 0.644, respectively.

**Conclusions:**

The DCFI and CFI may be potent indicators in predicting the use of STO in unilateral Crowe IV DDH. The optimal threshold for CFI and DCFI were 3.28 and 1.44 and had good sensitivity and specificity for predicting the use of STO during THA.

## Graphical abstract

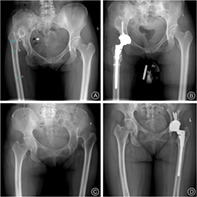

The diaphyseal canal flare index and canal flare index may be potent indicators in predicting the use of subtrochanteric osteotomy in unilateral Crowe IV developmental dysplasia of the hip.

## Introduction

Total hip arthroplasty (THA) with unilateral high dislocated hip is a technically demanding procedure in terms of preparation of the true acetabulum and reconstruction the equality of the leg length^1^, ^2^. THA can be performed in that situation if combined with femoral osteotomy after placement of the acetabular shell in the anatomy position^3^, ^4^, ^5^. Excessive lengthening of the leg may result in neurovascular damage that could be avoided by subtrochanteric osteotomy (STO)^6^. Cementless femoral prosthesis with STO, rather than a proximal femoral shortening osteotomy with distal advancement of the greater trochanter to restore the abductor muscle function, has been applied as it could avoid the risk of fibrous union of the greater trochanter^7^. However, some studies also reported that Crowe type IV developmental dysplasia of the hip (DDH) did not need the femoral osteotomy to reduce the hip to the true acetabulum^8^, ^9^, ^10^. Therefore, there is controversy about the application of osteotomy during THA in Crowe type IV DDH.

The shape of the femoral medullary canal is highly variable in high dislocated hip^11^, ^12^. The medullary canal is often small and can be unusually shaped, with a much smaller medial‐lateral than anterior–posterior diameter. Liu *et al*.^13^ revealed the narrowing mainly occurred at the metaphyseal and the proximal diaphyseal levels (the segment around lesser trochanter). The greater trochanter is located posteriorly and the proximal femur had more anteversion. Therefore, the variation of the femur in high dislocated hip mainly occurred at the metaphyseal and proximal diaphyseal levels. The different stress patterns and different soft tissue condition around the hip in high dislocated hip will result in different femoral proximal medullary canal morphology, and influence the decision of STO. A study found the absence of false acetabulum may be a potent indicator in predicting the use of STO in Crowe type IV DDH^14^. Sun *et al*.^15^ also revealed that indicator of dislocation height was useful in predicting the use of STO during THA. However, no study analyses the relation between femoral proximal morphology and STO in THA for Crowe type IV DDH.

The false acetabulum may be an influence factor in predicting the use of STO. The presence of a false acetabular stimulated the development of the proximal femur in Crowe IV DDH. Therefore, the femoral proximal medullary morphology may be a potent indicator in predicting the use of STO. The aims of this study were: (i) to investigate the canal flare index (CFI), metaphyseal canal flare index (MCFI) and diaphyseal canal flare index (DCFI) in unilateral Crowe type IV DDH on anteroposterior hip radiograph; (ii) to analyze the difference MCFI between the STO patients and the non‐STO patients; and (iii) to analyze the predictive values of the CFI, MCFI and DCFI for the use of STO.

## Patients and Methods

### Inclusion and Exclusion Criteria

The inclusion criteria were: (i) adults patients with unilateral Crowe type IV DDH; (ii) patients who underwent THA by a single surgeon in our institution; (iii) patients divide into two groups based on whether STO or not during THA; (iv) outcomes were to analyze the predictive values of the CFI, MCFI and DCFI for the use of STO; and (v) retrospective study. The exclusion criteria included: (i) patients with a history of pelvis and hip surgery, severe hip osteoarthritis or DDH of contralateral side, residual DDH (infection and trauma), knee flexion deformity or severe knee osteoarthritis; (ii) patients with history of cerebral palsy and poliomyelitis; and (iii) patients with a proximal cone shaped sleeve at the femoral side.

### Patients

A retrospective analysis was performed of all patients with unilateral Crowe type IV DDH undergoing THA surgery in our hospital, between April 2008 and June 2019. A total of 94 patients with unilateral Crowe type IV DDH were investigated. The study protocol was approved by the institution review board of our hospital (S2020‐138‐01). Informed consent was obtained from all the patients enrolled in the study.

### Groups According to the Subtrochanteric Osteotomy

The patients were divided into two groups according to the STO. In STO group, which consisted of 59 hips, the STO was performed during the THA. In non‐STO group, which consisted of 35 hips, the STO was not performed.

### Surgery

#### Anesthesia and Position

All patients were performed by a single surgeon (Y.G. Zhou) under general anesthesia in the lateral decubitus position.

#### Approach and Exposure

The procedure had been described in detail in our previous studies^3^, ^15^, ^16^. A posterolateral approach was used in all patients. The capsule was removed to expose the true acetabulum.

#### The Position of the Acetabular Shell

In order to use ceramic on ceramic bearing, the shell (size range 44–46 mm) was implanted at the level of the true acetabulum by reaming acetabulum posteriorly and inferiorly^3^. The acetabular shell was fixed by two screws.

#### Subtrochanteric Osteotomy

Gluteus minimus and gluteal sling release, and iliopsoas tenotomy were performed. If hip reduction with a femoral trial stem was impossible, a STO would be performed for femoral shortening. The position of osteotomy was located at the distal end of sleeve. The osteotomy length equaled the distance between the true acetabular center and femoral head center during the trial reduction minus 15 mm.

#### The Implant of the Sleeve and Femoral Stem

**Fig. 1:**
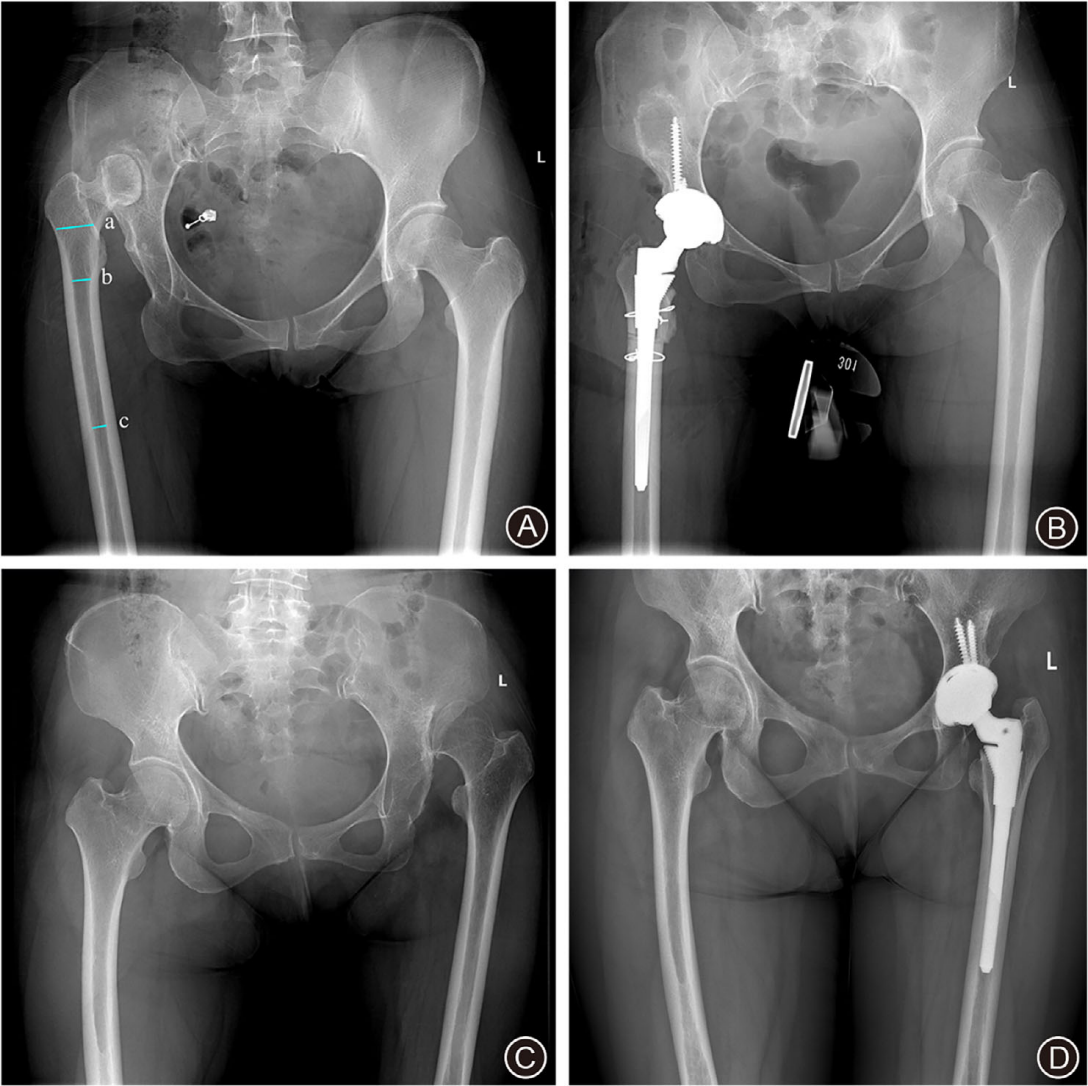
A 36‐year‐old female with right Crowe type IV DDH (A and B) and a 50‐year‐old female with left Crowe type IV DDH (C and D). (A) The preoperative AP hip radiograph (CFI: 2.81, MCFI: 2.44, DCFI: 1.15). (B) The postoperative radiograph 2 days after right hip surgery. THA with STO was performed. (C) The preoperative AP hip radiograph (CFI: 4.25, MCFI: 2.24, DCFI: 1.89). (D) The postoperative radiograph 2 days after right hip surgery. THA without STO was performed. a: 20 mm above the center of lesser trochanter (CLT), b: 20 mm below the CLT, c: isthmus.

Prophylactic cerclage wires were placed both proximally and distally around the location of the osteotomy. A sleeve that was based on the proximal femoral intramedullary morphology was implanted. And a femoral stem was implanted based on the femoral intramedullary canal. The Pinnacle acetabular shell, Biolox delta ceramic on ceramic bearing, a S‐ROM femoral stem with proximal sleeve (DePuy, Warsaw, Indiana, USA) were used in all patients (Fig. 1).

### Radiographic Measurement

A standard anteroposterior hip radiograph was obtained preoperatively. All the radiographs were viewed and measured on a picture archiving and communication system (PACS, UniWeb Viewer, version 4.0, EBM technologies, Taiwan, China). All radiographic measurements were analyzed twice, each by two investigators who had not participated in the surgery. The actual values for each measurement were obtained by calibration using the known diameter of the ceramic femoral head or marker for references. The following parameters were measured: The widths of medullary canals were measured at 20 mm above the center of lesser trochanter (CLT)，20 mm below the CLT and the isthmus^17^, ^18^.

#### Canal Flare Index

CFI was calculated as the ratio of the widths of medullary canal at the level of the 20 mm above the CLT to the widths of medullary canal at the isthmus^19^, which was used to describe the medullary canal morphology of the proximal femur.

#### Metaphyseal Canal Flare Index

MCFI was calculated as the ratio of the widths of medullary canal at the level of 20 mm above to 20 mm below the CLT^20^, which was used to describe the medullary canal morphology of the proximal metaphyseal femur.

#### Diaphyseal Canal Flare Index

DCFI was calculated as the ratio of the widths of medullary canal at the level of the 20 mm below the CLT to the widths of medullary canal at the isthmus^13^. which was used to describe the medullary canal morphology of the proximal diaphyseal femur.

#### Intertrochanteric Distance

The intertrochanteric distance was defined as the vertical distance from the apex of greater trochanter to the CLT^21^.

### Statistical Analysis

The intraclass correlation coefficient (ICC) was used to determine the variations of the different measurements. Categorical data were compared using a chi‐squared test. The independent‐samples *t* test or Mann–Whitney U test was used to compare continuous data between the STO and non‐STO groups. Receiver operating characteristic (ROC) curves were generated to determine the value of each measurement. The area under the curve (AUC) was calculated. The discriminatory value of curves was interpreted as excellent (0.9 to 1), good (0.8 to 0.89), fair (0.7 to 0.79), poor (0.6 to 0.69), or as failing or having no discriminatory capacity (0.5 to 0.59). All tests were performed using SPSS (version 26 for Mac; IBM Corp., Armonk, NY, USA). A *P*‐value <0.05 was considered significant in all analysis. The figures were drawn by GraphPad Prism software (version 8.4.0 for Mac; GraphPad software, San Diego, CA, USA).

## Results

### General Results

The characteristics of the patients were shown in Table 1. After dividing the two groups according to STO, we found that there was a statistical difference in side (*P* = 0.001) and there were no statistical differences in gender, age, and body mass index.

**TABLE 1:** Demographics and measurement parameters between the STO and non‐STO groups

Parameter	STO (n = 59)	Non‐STO (n = 35)	*P* values
Gender (female:male, %)	56 (95): 3 (5)	33 (94): 2 (6)	0.895
Age (years)	38.1 ± 12.0	39.4 ± 10.1	0.484
BMI (kg/m^2^)	22.2 ± 2.8	23.5 ± 3.7	0.180
Side (right: left, %)	37 (63): 22 (37)	9 (26): 26 (74)	0.001
CFI	2.8 ± 0.6	3.9 ± 0.9	<0.001
MCFI	2.3 ± 0.5	2.4 ± 0.5	0.204
DCFI	1.3 ± 0.2	1.7 ± 0.3	<0.001
Intertrochanteric distance (mm)	54.2 ± 6.3	50.2 ± 5.2	0.004

BMI, Body mass index; CFI, Canal flare index; DCFI, Diaphyseal canal flare index; MCFI, Metaphyseal canal flare index; STO, Subtrochanteric osteotomy.

### Radiographic Measurement

The CFI and DCFI in the STO group were lower than those in non‐STO group (*P* < 0.001). However, there was no statistical difference in MCFI between the two groups (*P* = 0.204). The intertrochanter distances in STO group were higher than that in non‐STO group (*P* = 0.004) (Table 1).

### The Predictive Values of the CFI, MCFI and DCFI for the use of STO

**Fig. 2:**
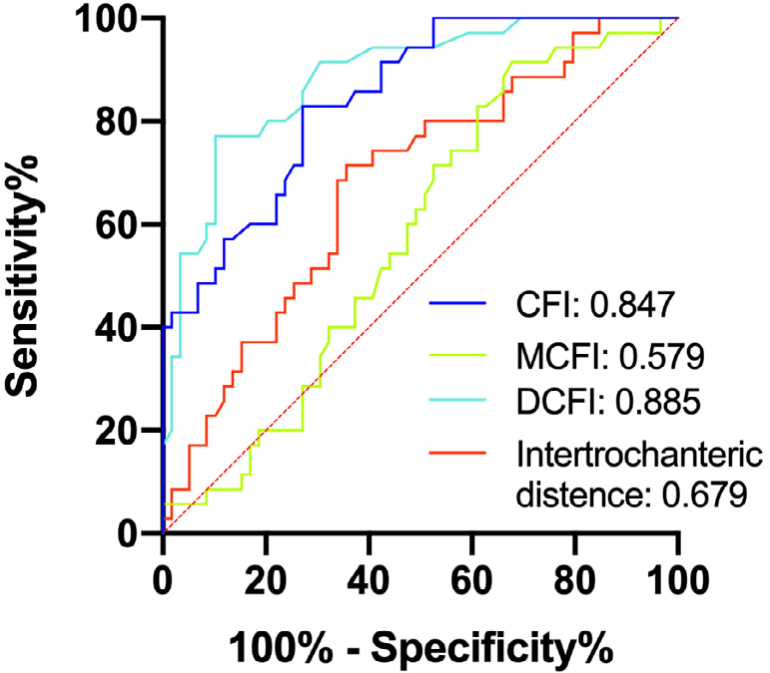
The ROC curves for predicting the use of STO in unilateral Crowe IV DDH. The CFI (AUC: 0.847) and DCFI (AUC: 0.885) had good predictive value.

The ROC curves shown that DCFI had the highest AUC, at 0.885. This was followed by the CFI, which had an AUC of 0.847. This AUCs of these two parameters were all between 0.8 and 0.9, indicating that they had good performance for predicting the use of STO in THA for Crowe type IV DDH. In contrast, the intertrochanteric distance had an AUC of 0.679, which was the second lowest value and was only slightly higher than the AUC of MCFI count at 0.579. The AUCs of these two parameters were both lower than 0.7, indicating that they had poor predictive value (Fig. 2).

Based on our data, the optimal threshold for DCFI was 1.44, which lead to a sensitivity, specificity, positive predictive value (PPV), and negative predictive value (NPV) of 0.771, 0.898, 0.869, and 0.818, respectively. For CFI, the optimal threshold was 3.28, resulting in a sensitivity, specificity, PPV, and NPV of 0.829, 0.729, 0.878, and 0.644, respectively.

### The Intraclass Correlation Coefficient

Both the intraobserver and the interobserver agreement were found to be nearly perfect for all of the measurements (Table 2).

**TABLE 2:** Intraobserver and interobserver variations of measurements

Variable	Intraobserver (ICC)	Interobserver (ICC)
CFI	0.84	0.83
MCFI	0.87	0.87
DCFI	0.90	0.89
Intertrochanteric distance	0.85	0.85

CFI, Canal flare index; DCFI, Diaphyseal canal flare index; ICC, intraclass correlation coefficient; MCFI, Metaphyseal canal flare index.

## Discussion

This is the first study on the influence of femoral proximal morphology on STO in total hip arthroplasty for unilateral Crowe type IV DDH. In our study, we select four parameters, including CFI, MCFI, DCFI and intertrochanteric distance, to represent the femoral proximal morphology, and found the DCFI and CFI had good performance for predicting the use of STO in THA for Crowe type IV DDH.

### The Anatomical Abnormalities of Proximal Femurs in DDH

The anatomical abnormalities of proximal femurs caused by DDH make THA particularly difficult to perform. The intramedullary femoral canal had reduced mediolateral and anteroposterior dimensions in DDH patients^17^. Noble *et al*.^22^ found the primary anatomical abnormality of the dysplastic femur was rotational, and the rotational abnormality occurred in the diaphysis between the lesser trochanter and the isthmus and was not attributable to torsional abnormalities of the metaphysis. Liu *et al*.^13^ reported that Crowe IV DDH had a dramatic change in the femoral proximal morphology, especially the dramatic narrowing of medullary canal around the level of the lesser trochanter. The false acetabulum or osteoarthritis secondary to Crowe IV DDH may promote the development of the proximal femur^11^. Therefore, the femoral proximal medullary morphology in Crowe IV DDH may be associated with different loading patterns and different soft tissue condition, which eventually affected reduction during THA.

### The Subtrochanteric Osteotomy was Performed during THA

One of the difficulties during THA for Crowe type IV DDH is to safely reduce the hip to the true acetabulum and protect the neurovascular structures^23^. Femoral osteotomy was widely used during THA in reduction of the hip, including proximal osteotomy and STO. The most commonly used method of femoral osteotomy is STO during THA in which transverse osteotomy is made distal to the lesser trochanter of the femur^24^. Several authors had reported successful long‐term results of THA with STO in Crowe type IV DDH^24^, ^25^, ^26^. However, not all the Crowe type IV DDHs need the femoral osteotomy to reduce the hip to the true acetabulum. Lai *et al*.^8^ were able to safely place the acetabular cup at the level of anatomical position without femoral osteotomy for unilateral Crowe type IV DDH by iliofemoral distraction before THA. Some techniques, such as intravenous injection of rocuronium combined with continuous strong traction^10^ and direct leverage^27^, were reported to be able to achieve hip reduction without femoral osteotomy. Some factors were also reported to be able to achieve hip reduction without femoral osteotomy, such as the false acetabulum before THA or osteoarthritis secondary to Crowe IV DDH and the height of dislocation^11^, ^14^, ^16^.

### The Influence of Femoral Proximal Medullary Morphology on STO in THA

Therefore, there was controversy about the application of osteotomy during THA. And there was no study to discuss the influence of femoral proximal medullary morphology on STO in THA for unilateral Crowe IV DDH. In our study, the CFI and DCFI in the STO group were lower than those in non‐STO group. However, there was no statistical difference in MCFI. The proximal femur in STO group was stovepipe shaped, with a smaller CFI (2.8 ± 0.6), compared with that in non‐STO group (3.9 ± 0.9). The abnormality of femoral proximal medullary morphology in the two groups arise within the diaphysis between the 20 mm below the CLT and the isthmus. The ROC curves also shown CFI and DCFI had good performance for predicting the use of STO in THA for Crowe type IV DDH. The optimal threshold for CFI and DCFI was 3.28 and 1.44, respectively. And MCFI had poor predictive value. In another study, there was no significant difference in MCFI between the Crowe IVA (with no false acetabular) and the Crowe IVB (with a false acetabular) DDH. However, there was a significant difference in CFI and DCFI between the two Crowe IV DDHs^28^. The false acetabular stimulated the development of the proximal femur in Crowe IV DDH. Therefore, the presence of the false acetabular may be an important factor determining STO application^14^, ^29^. Tamura *et al*.^21^ found the intertrochanteric distance was a morphological factor related to femoral‐length asymmetry in unilateral osteoarthritis secondary to DDH. We also analyzed the influence of the intertrochanteric distance on STO and found it had poor predictive value. Sun *et al*.^15^ revealed that indicator of dislocation height was useful in predicting the use of STO during THA for Crowe unilateral Crowe IV DDH. Our previous research also found the use of a cone shaped sleeve combined with trochanteric osteotomy may effectively reduce the application of STO, and STO could increase the risk of postoperative dislocation^29^.

### Limitations of the Study

There are several limitations in this study. First, this was a retrospective study. Second, all measurements were based on the plain radiographs not the CT and three‐dimensional computer reconstruction models. Third, only one single femoral component (a S‐ROM femoral stem with proximal sleeve) was used in all patients. The results may not be applicable to other types of femoral component. Finally, the long‐term results of this study may need further observation between the two groups.

### Conclusions

The current study reveals that the DCFI and CFI may be potent indicators in predicting the use of STO in unilateral Crowe IV DDH. The optimal threshold for CFI and DCFI was 3.28 and 1.44 and had good sensitivity and specificity for predicting the use of STO during THA.
